# A novel reconstruction model for thoracic spinal cord injury in swine

**DOI:** 10.1371/journal.pone.0308637

**Published:** 2024-09-26

**Authors:** Ali Nourbakhsh, Catherine Takawira, Elise Barras, Chiara Hampton, Mariano Carossino, Khoivu Nguyen, Lorrie Gaschen, Mandi J. Lopez

**Affiliations:** 1 OrthoAtlanta, Stockbridge, Georgia, United States of America; 2 Department of Veterinary Clinical Science, Louisiana State University School of Veterinary Medicine, Baton Rouge, Louisiana, United States of America; 3 University of Florida, Gainesville, Florida, United States of America; 4 Department of Pathobiological Sciences, Louisiana State University School of Veterinary Medicine, Baton Rouge, Louisiana, United States of America; 5 Department of Large Animal Clinical Sciences, College of Veterinary Medicine, University of Tennessee, Knoxville, Tennessee, United States of America; University of Rijeka Faculty of Medicine: Sveuciliste u Rijeci Medicinski fakultet, CROATIA

## Abstract

Spinal cord (SC) reconstruction (process to reestablish the severed neural continuity at the injury site) may provide better recovery from blunt SC injury (SCI). A miniature swine model of blunt SC compression was used to test the hypothesis that reconstruction of the SC with sural nerve in combination with surgical decompression and stabilization improves functional, macro- and microstructural recovery compared to decompression and stabilization alone. Following blunt T9-T11 SC compression injury, five adult Yucatan gilts randomly received laminectomy and polyethylene glycol (as fusogen) with (n = 3) or without (n = 2) sural nerve graft SC reconstruction. Fusogens are a heterogeneous collection of chemicals that fuse the axon membrane and are currently used to augment epineural coaptation during peripheral nerve graft reconstruction. Outcome measures of recovery included weekly sensory and motor assessments, various measurements obtained from computed tomography (CT) myelograms up to 12 weeks after injury Measurements from postmortem magnetic resonance imaging (MRI) and results from spinal cord histology performed 12 weeks after injury were also reported. Vertebral canal (VC), SC and dural sac (DS) dimensions and areas were quantified on 2-D CT images adjacent to the injury. Effort to stand and response to physical manipulation improved 7 and 9 weeks and 9 and 10 weeks, respectively, after injury in the reconstruction group. Myelogram measures indicated greater T13-T14 VC, smaller SC, and smaller DS dimensions in the reconstruction cohort, and increased DS area increased DS/VC area ratio, and higher contrast migration over time. Spinal cord continuity was evident in 2 gilts in the reconstruction cohort with CT and MRI imaging. At the SCI, microstructural alterations included axonal loss and glial scarring. Better functional outcomes were observed in subjects treated with sural nerve SC reconstruction. Study results support the use of this adult swine model of blunt SCI. Long-term studies with different nerve grafts or fusogens are required to expand upon these findings.

## Introduction

Spinal cord injuries (SCI) are devastating injuries that affect multiple physiological functions. The rate of thoracolumbar fracture in patients from blunt trauma is 6.9% with an associated SCI rate of 26.6% [1]. A 1 or 2 grade improvement on the American Spinal Injury Association scale does not equate to functional recovery, and less than 20% of thoracic SCIs improve ≥ 1 grade [2]. As such, the injuries impose immediate and long term burdens on the healthcare system including intensive care, spinal and neurogenic shock treatment, surgical spinal stabilization, and chronic conditions like respiratory insufficiency, decubitus ulcers, limb contracture, incontinence, paresis, paralysis, and challenges with activities of daily living. The most common types of spinal cord injuries happen after falls and accidents or gunshot wounds causing vertebral fracture and subsequent injury of the spinal cord by the bone fragments. Complete spinal cord injury is the absence of sensory and motor function below the injury level [3]. Incomplete injuries are preservation of sensory with or without motor function below the neurologic injury level. The most important prognostic factor for functional improvement in SCI is the initial neurological status after the injury [4]. For complete SCI the rate of improvement is 2.5% as opposed to 33–100% for incomplete SCIs [5–10].

The current standard of care includes decompression and stabilization at the level of the injury [11]. Decompression techniques include removal of the bone fragments and parts of the vertebrae including lamina and stabilization is performed through multiple methods of instrumentation including pedicle screw fixation. The use of nerve auto- and allografts to reconstruct peripheral nerve injuries is well established. Specifically, this technique restores continuity between healthy nerve tissue on either side of a neural injury with a nerve graft, typically with a superficial sensory nerve such as the sural or lateral antebrachial nerve [12]. Some tissue engineering strategies to restore damaged spinal cord axons include synthetic biomaterial implants with channels oriented parallel to spinal cord tracts, and they are reported to have some success in animal models [13]. However, there are compelling benefits of viable tissue autografts over synthetic implants, including lack of immunogenicity, native architecture, and viable progenitor cells [14]. Peripheral nerve grafts to facilitate neuroregeneration and neuron and axon replacement within funiculi of damaged spinal tracts, however, have not been extensively evaluated in large animal models of common SCIs with low recovery rates [15–17].

The majority of SCI investigations using animal models, including murine and porcine, focus on transection of the spinal cord rather than blunt contusion [18, 19]. Such injuries are not as common as blunt injuries in human patients, additionally transection of the spinal cord has less severe vascular and axonal damage and higher incidences of spontaneous neurological recovery than blunt injuries [19]. The Brown-Séquard syndrome is an example of sharp spinal cord hemitransection, and it has the best recovery potential among all spinal cord injuries [18]. Blunt injuries of the spinal cord as seen in other spinal cord injury syndromes have a much lower prognosis for recovery. Large animal spinal cord size and neuroanatomy are closer to humans than lapin and rodent. The larger size also permits use of standard human surgical techniques and instrumentation and allows for evaluation of novel translational techniques like sural nerve spinal cord reconstruction size [20].

Fusogens like polyethylene glycol (PEG), poloxamers, and poloxamines, are a heterogeneous collection of chemicals that fuse the axon membrane and are currently used to augment epineural coaptation during peripheral nerve graft reconstruction [21–23]. Polyethylene glycol, the most extensively studied and commonly used fusogen, functions via cell aggregation, and membrane modification, and destabilization [24]. Specifically, as a hydrophilic compound, PEG brings cells into close apposition by removing water between them while altering membrane surface charges to reduce repulsion to bind the membrane surface at the lipid-head cohort. Ultimately, the combination of cell aggregation and membrane destabilization permits cell membrane fusion [25]. Wojcieszyn et al. studied the mechanism of PEG mediates cell fusion by examining the cytoplasmic markers and movements of membrane lipids and proteins from erythrocytes that have been fused to cultured cells. They found that with the presence of PEG membranes are approximated closely. Additionally the lipid probes transfer quickly from the membranes of erythrocytes to cultured cells with PEG which is truly indicative of fusion [26]. Current knowledge supports that PEG-fusion of single transection or ablation-type peripheral nerve injuries improves morphological, functional, and behavioral recovery, and application of PEG immediately following sharp transection of the spinal cord is reported to improve recovery in numerous species including dogs and rats, and after blunt trauma in guinea pigs [27–29]. In a thoracic SCI model Tabakow et al. reported the use of autologous stem cells combined with sural nerve spinal cord reconstruction [30]. Information is limited about the potential effects of PEG combined with reconstruction using autologous peripheral nerve tissue in models of blunt SCI.

The primary objective of this study was to test the effect of sural nerve graft with PEG versus PEG alonefollowing standard surgical decompression and stabilization on the functional and radiological outcome in an adult miniature swine model of blunt SCI. To test this hypothesis, SCI was induced after laminectomy in five adult Yucatan miniature gilts following a model of blunt thoracic SCI previously established in immature domestic gilts [18]. After laminectomy, gilts were randomly assigned to receive PEG and spinal cord reconstruction with autologous sural nerve graft or PEG alone. The secondary objective of this study was to establish an adult porcine model of blunt thoracic SCI treated with autologous sural nerve reconstruction as a platform on which to expand this important area of research.

## Materials and methods

### Animals and study design

Procedures were approved by the Institutional Animal Care and Use Committee (IACUC, protocol #19–080) prior to the start of the experiment. Five Yucatan gilts (*Sus scrofa domestica*, 9.4 ± 0.5 months, 47.9 +/- 6.4 kg, mean ± SEM) from a pathogen free facility (Sinclair Bio Resource, LLC) were housed and cared for in an Association for Assessment and Accreditation of Laboratory Animal Care (AAALAC) International accredited facility in accordance with the Guide for the Care and Use of Laboratory Animals [31]. The Yucatan minipig is the most commonly utilized strain in spinal cord injury (SCI) research, with the Gottingen mini, Vietnamese potbellied mini, and Yorkshire pigs following behind. The Yucatan pig, weighing around 70 kg, is known for being hairless and docile. It’s also an inbred strain, which results in reduced genetic variability. This aspect is crucial when examining genetic alterations in SCI research and evaluating reactions to novel drug treatments [32]. The Yucatan pig is more suitable for long-term research studies. They grow at a slower pace, typically gaining 3–5 kg per month [33]. During a 14-day acclimation period prior to surgery, gilts were housed in cohorts and offered a pelleted diet (Mazuri^®^ Mini Pig Active Adult #5Z9, Land O’Lakes, Inc, Saint Paul, MN) twice daily with unrestricted water access. After surgery, gilts were individually housed side by side in 4.5 m^2^ pens on fluid resistant, padded mats where intravenous fluid support and urinary catheters could be maintained as medically necessary, with body repositioning being performed every 3–4 hr until gilts could do so independently. Complete blood counts and serum chemistries were performed immediately prior to surgery as well as 6 and 12 weeks post-operatively. Medical care of the gilts was provided by licensed veterinarians for the duration of the study. On the day of the surgical procedure, pigs were randomly assigned to one of two cohorts: a control cohort receiving SCI and PEG (DPEG; n = 2), and a treatment cohort receiving SCI, and a sural nerve autograft for reconstruction followed by PEG application (DRPEG; n = 3). Blunt SCI was induced at T10 (based on preoperative CT scan performed with a tattoo placed on the skin as a markers) under general anesthesia following dorsal laminectomy. Thoracic computed tomography (CT) myelograms were performed prior to and to follow surgery, and 6 and 12 weeks postoperatively. Various measurements including sagittal (height) and transverse (width) diameters of the vertebral canal and SC were performed at, and cranial and caudal to the injury site. Gilts were assessed weekly using a modified porcine thoracic injury behavior scale [10]. Magnetic resonance imaging was performed post-mortem on formalin fixed thoracic spine sections. Spinal microstructure, including gray and white matter were quantified on transverse sections at the level of the injury with light microscopy.

### Anesthesia

All anesthetic procedures were planned and performed by a board-certified veterinary anesthesiologist (CEH) according to IACUC approved procedures and as previously reported [34]. Gabapentin (10 mg/kg, PO; Time-Cap Labs, Inc., Farmingdale, NY) was administered the evening prior to and the morning of surgery to provide mild tranquilization. A combination of tiletamine-zolazepam (3.3 mg/kg; Telazol^®^, Zoetis, Kalamazoo, MI), atropine (0.04 mg/kg; Atropine Sulfate, VetOne, Boise, ID USA), and morphine (1 mg/kg; morphine sulphate; Hospira, Lake Forest, IL) was administered intramuscularly for pre-anesthetic sedation. Induction of general anesthesia was performed by delivering isoflurane (VETONE^®^, Fluriso^™^, MWI, Boise, ID) via face mask to achieve sufficient muscle relaxation to perform endotracheal intubation. Adequate anesthetic depth throughout the diagnostic and surgical procedures was maintained with isoflurane carried variably in oxygen (97–99%) via a breathing circuit connected to a circle system.

Catheters (20G, 1”; Surflash^™^ polyurethane IV catheter, Somerset, NJ) were placed in the caudal auricular vein for fluid (Lactated Ringer’s solution, 5 mL/kg/hr, IV; Vetivex^®^, Dechra Veterinary Products, Overland Park, KS) and drug administration, and in the cranial auricular artery or the coccygeal artery (22-24G, 1”; Surflash^™^ Polyurethane IV Catheter, Somerset, NJ) for invasive blood pressure monitoring. Cefazolin (22 mg/kg; Cefazolin for Injection, USP, West-Ward Pharmaceutical Corp., Eaton Town, NJ) was administered intravenously via the auricular vein catheter following intubation and subsequently every 90 minutes. Monitoring of heart rate and rhythm, invasive arterial blood pressures, respiratory rate, core body temperature, and peripheral oxygen saturation was performed throughout the anesthetic period. HR and cardiac rhythm were monitored using electrocardiography and a multiparameter monitor (Spectrum, Datascope Corp, Mahawah, NJ), hemoglobin oxygen saturation was monitored using pulse oximetry (SpO2, Masimo Technology, Irvine, CA), and BT was monitored using a thermistor probe placed in the lower third of the esophagus. SAP, DAP, and MAP were monitored by connecting a calibrated blood pressure transducer (DTXPlus, BD Medical Systems, Sandy, UT) zeroed and leveled to the sternum of the pig to an arterial catheter aseptically placed in the coccygeal artery. A gas analyzer (Gas Module SE, Datascope, Mindray North America, Mahwah, NJ) was used to monitor expired partial pressure of carbon dioxide (EtCO2), and anesthetic agent (EtISO). Blood gas analysis (EPOC Blood Analysis System, Siemens Medical Solutions USA, Malverne, PA) was performed on arterial blood samples collected anaerobically to monitor plasma electrolytes, gases, and metabolic parameters [34].

Mechanical ventilation was initiated immediately prior to the start of the surgical procedure to provide adequate alveolar ventilation (target EtCO_2_ = 35–45 mm Hg) and a stable anesthetic plane. Loading doses of fentanyl (5 μg/kg; fentanyl citrate, Hospira, Lake Forest, IL), ketamine (0.5 mg/kg; Zetamine, Vet One, Boise, ID) and lidocaine (2 mg/kg; Lidocaine, VetOne, Boise, ID) were administered over 5 minutes prior to the start of the correspondent constant rate infusions (CRIs) of fentanyl (25 μg/kg/hr) or morphine (0.5 mg/kg/hr), ketamine (20 μg/kg/min), and lidocaine (50 μg/kg/min). Additional doses of fentanyl (5 μg/kg) and/or lidocaine (1 mg/kg) were administered intravenously as needed. Fifteen minutes prior to the dorsal laminectomy, cis-atracurium (0.1–0.2 mg/kg IV; Cis-atracurium besylate injection, Sandoz Inc., Princeton, NJ) was administered to obtain muscle relaxation, with subsequent doses administered as needed. A transurethral urinary catheter (5-8F, Female Canine Guidewire Inserted Foley Catheter Kit, MILA International Inc., Florence, Kentucky) was placed during general anesthesia and urine production measured during the operative and post-operative periods it remained in place. A warm air blowing device (Bair Hugger, Arizant Inc., Eden Prairie, MN) and a circulating water blanket (T/Pump TP500, Gaymar Industries, Inc., Orchard Park, NY) were used to maintain body temperature between 36.6 and 38.3 °C.

### Sural nerve graft harvest

**Fig 1:**
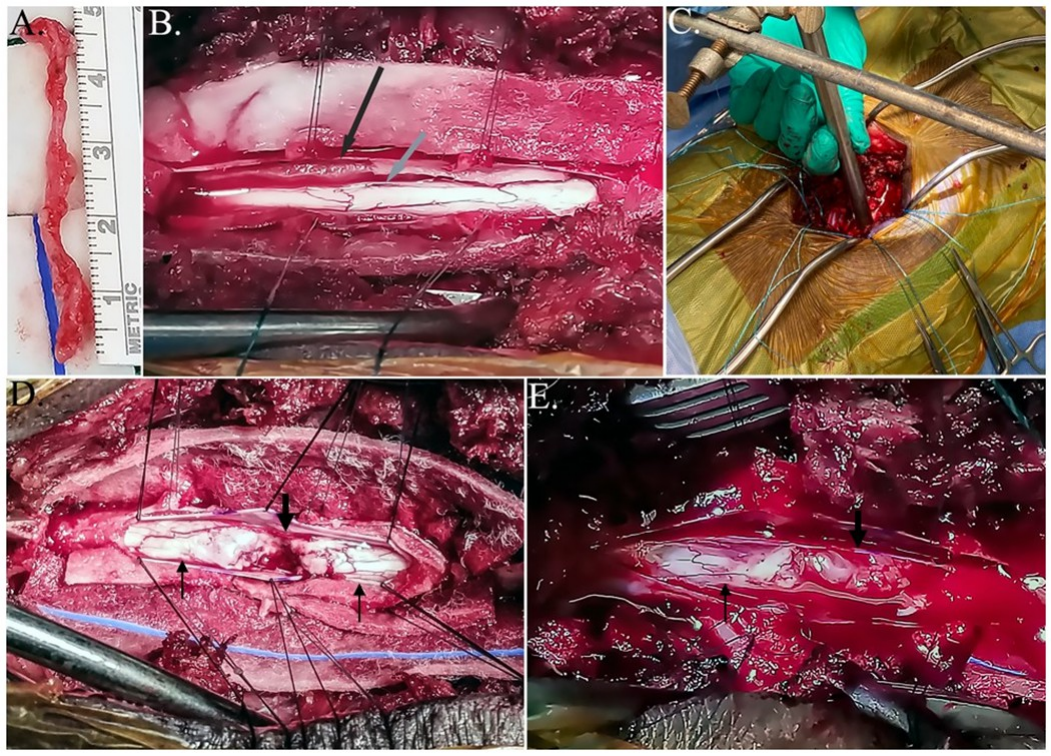
Photographs of sural nerve graft (A); Exposed spinal cord with dural (black arrow) and peridural (gray arrow) membranes indicated (B); Column containing impactor rod positioned over exposed spinal cord (C); Spinal cord injury (D); and sural nerve graft within a spinal cord defect (E). Black vertical thin arrows point to the intact spinal cord and the black vertical thick arrows point to the area of spinal cord injury (D) and sural nerve (E).

With gilts in lateral recumbency, the skin of the rear limb was aseptically prepared similarly to that of the dorsal spine, the surgical site isolated with surgical drapes, and the sural nerve was exposed using a combination of blunt and sharp dissection as previously described [35]. Briefly, the skin and subcutaneous tissue were incised along the caudolateral aspect of the hind limb beginning at the proximal calcaneus. The sural nerve was gently dissected free of connective tissue from its location immediately adjacent to the small saphenous vein [36], and 4–5 cm of nerve resected (Fig 1A). The nerve graft was maintained in sterile NaCl 0.9% (USP, Baxter Healthcare Corporation, Deerfield, IL) at the room temperature until implantation. Fascial and subcutaneous tissues as well as skin were then closed routinely. Sural nerve graft harvest and the spinal cord procedure were done in the same setting.

### Spinal surgery and cord injury

With gilts in sternal recumbency, the surface of the skin over the dorsal spine was clipped, cleansed with 2% chlorhexidine (VetOne ^®^, MWI, Boise, ID), and aseptically prepared with alternating 2% chlorhexidine/70% isopropanol scrubs. Following isolation of the surgical field with sterile drapes, a standard dorsal surgical approach was made to the T9-T11 spine while hemostasis was maintained with electrocautery. The dorsal spinous processes were exposed with a combination of sharp and blunt dissection and T9-T11 laminectomies performed manually. Epidural adipose tissue was gently extracted to expose the dura. The dural and peridural membranes were sharply incised, retracted with nylon suture (#4–0 Nurolon^®^, Ethicon Inc., San Angelo, TX), and tensioned with Halstead mosquito forceps to expose the spinal cord (Fig 1B).

A sterile device constructed according to a published report was used to induce blunt injury (Fig 1C) [18]. Specifically, a hollow aluminum column (1 cm inner diameter, 50 cm length) was stabilized vertically to and 2 cm above the T10 exposed spinal cord using a clamp extending horizontally from a 0.5 cm vertical rod threaded into a 15 cm x 10 cm metal base placed parallel to the thorax on the surgery table surface. The device was fixed to the table. A solid 100 g rod (1 cm diameter, 25 cm length) was dropped within the column onto the exposed spinal tissue from 50 cm height using the length of the hollow cylindrical device and maintained in place for 5 consecutive minutes. The rod was then gently elevated, and the device was removed from the surgical field.

The cranial and caudal edges of the injury were sharpened, and the damaged neural tissue carefully removed without disturbing any intact tissue (Fig 1D). The loose fragments of the neural tissue with no or minimal connection to the cord were removed. For cord reconstruction, the sural nerve was sharply transected into three segments the length of disrupted SC and placed into the defect such that the ends contacted the refreshed edges of the damaged cord (Fig 1E). Subsequently, 0.5 mL of 30% PEG were applied to the surface of the nerve graft segments. The same volume of PEG was applied to defects without grafts.

The dural and peridural membranes were opposed with interrupted, #4–0 nylon sutures (Nurolon^™^, Ethicon Inc.,). The length of the laminectomy was stabilized on each side with 6- or 8-hole, 3.5 mm low contact, dynamic compression plates (Depuy Synthes, West Chester, PA) with 3.5 mm diameter, 8–12 mm long pedicle screws. Following copious lavage with sterile saline, tissues were opposed in a layered closure with #0 polydioxanone suture (Securodox *PLUS*^™^, Riverpoint Medical, Portland, OR) in a simple continuous pattern for fascial and subcutaneous tissue and #2 nylon (Ethilon^™^, Ethicon Inc., San Angelo, TX) in interrupted horizontal mattress sutures for skin. A betadine impregnated betadine adhesive drape (3M^™^Ioban^™^, 3M Healthcare, St. Paul, MN) was applied over the incision.

### Post-surgery care

Supportive care and fluid management were performed postoperatively. Xylazine (0.2–0.5mg/kg q1 to 2 hr PRN) and midazolam (0.1–0.6mg/kg q1 to 2hr PRN) as needed during an overnight stay in ICU. Gilts were monitored every 1–3 hr for the first 7 days post-surgery and pain was managed with intravenous morphine (0.5 mg/kg/hr) or fentanyl (25 μg/kg/hr) with lidocaine (50 μg/kg/min) and ketamine (20 μg/kg/min) in all subjects for the first 12 hr post laminectomy. Hydration was maintained with lactated Ringers’ solution for 4–5 days at rate of 120 mL/hr until each gilt was able to drink voluntarily from a water bottle. Fentanyl transdermal patches (100ug patches) were applied to the lateral hock of the left or right hind limb and covered by elastikon immediately post op for 3 days. Animals received omeprazole (0.1 ml PO SID), enroflaxacin (2.5mg/kg IM SID) for 6 days, and gabapentin 20mg/kg PO BID) for 14 days beginning the day after surgery. Immediately post-surgery, gilts received (1mg/kg SC, Cerenia, 10mg/mL injectable, Pfizer), injectable carprofen (4mg/kg SC, Rimadyl (carprofen) 50mg/mL, Zoetis) was administered immediately following surgery and then oral carprofen (4mg/kg PO, OstiFen (carprofen), Vet One)) was given 4 days after that.

If gilts developed signs of urinary tract infection, antibiotic therapy was instituted based on aerobic bacterial culture results. Urine output was measured every 1–2 hr until removal of the urinary catheters which were replaced every 7–10 days and removed 14–21 days after surgery after reflexive voiding was confirmed. Gilts were repositioned every 2–3 hr until they could sit up and reposition themselves independently. Daily physical therapy consisting of 20 minutes of manual hind limb flexion and extension was initiated 3 days after surgery and continued throughout the study. Motor function and response to sensory stimulation was digitally recorded weekly beginning 7 days after surgery. For sensory stimulation, the knee joint of each hind limb was gently flexed for 3–5 seconds to observe retraction response. Observations were made to determine the sensitivity of responses elicited by this stimulation. Individuals unaware of treatment viewed the digital recordings in random order and provided numeric scores for sensory and motor function using a modification of a published rubric (Table 1), except for the response to physical manipulation, which was recorded weekly at the beginning of the physical therapy session. Blood was collected from the cranial vena cava for complete blood count and serum chemistry analysis with gilts anesthetized as described above immediately before as well as 6 and 12 weeks after surgery, with the latter two collections just prior to CT imaging. Briefly, in left lateral recumbency, the right forelimb was gently retracted to allow ease of access to the collection site. A 1.5 inch 18 gauge needle on a 20ml syringe was inserted at a 45 degree angle into the cranial vena cava about 1 inch cranial to the sternu, and 1 inch to the midline.

**Table 1:** Sensory and motor function assessment score.

Neurological Grading Scale		
**Response To Touch (Stifle/Calyx)**		
1	Sensory	No sensation. Animal does not react.
2		Impaired sensation. Aware of touch but no limb movement
3		Slight sensation. Visible limb movement in response.
4		Normal sensation.
**Hip Movement (Flexion/Extension)**		
1	Motor	No voluntary movement. Joint remains in either flexed or extended position.
2		Involuntary and random joint flexion or extension.
3		Resists join manipulation.
4		Voluntary extends joint when placed in a flexed weight bearing position.
5		Voluntarily flexes and extends joint in order to actively move around cage.
**Stifle Movement (Flexion/Extension)**		
1	Motor	No voluntary movement. Joint remains in either flexed or extended position.
2		Involuntary and random joint flexion or extension.
3		Resists join manipulation.
4		Voluntary extends joint when placed in a flexed weight bearing position.
5		Voluntarily flexes and extends joint in order to actively move around cage.
**Effort to Stand**		
1	Motor	Stands on front limbs. Rear limbs remain extended or tucked when lifted.
2		Sits up on the hind limbs with one or both legs tucked under the rump. Moves around the cage.
3		Sits up on one side of rump, able to lift part of rump. Attempts to “find the ground” when lifted.
4		Sits up, with both legs tucked under. Places rear limbs on ground. Attempts to bare weight on rear limbs when lifted.
5		Able to lift entire rump significantly using rear limbs.
**Rump Scooting in Seated Position**		
1	Motor	Remains seated.
2		Uses front limbs to pull weight around the cage.
3		Flexes and extends hind limbs occasionally to move small distances.
4		Actively flexes and extends hind limbs in a coordinated fashion while bearing weight on front limbs.
5		Only uses hind limbs to rump scoot.
**Body Position**		
1	Motor	Lying down flat.
2		Sitting on the hind limbs with both legs extended forward.
3		Sitting on the hind limbs with one leg extended forward and the other tucked under the rump. Unable to switch side of recumbence.
4		Sitting up on the hind limbs with both legs tucked under rump. Able to fully switch side of recumbence.
5		Able to lift rump up on own.
**Response to Physical Manipulation**		
1	Motor	No resistance observed.
2		Slight, inconsistent resistance observed.
3		Actively resists hind limb manipulation.
4		Actively retracts hind when manually extended.
5		Active hind limb movement including flexion and extension at the stifle and hip joint.

### Computed tomography (CT)

With gilts anesthetized as described above for anesthetic induction and maintenance, CT thoracic and lumbar myelograms were performed (GE LightSpeed, GE Hangwei Medical System, China) prior to and immediately, 6, and 12 weeks after surgery as previously described to assess the continuity of the spinal cord [37]. Briefly, gilts were placed in sternal recumbency with their coxofemoral and stifle joints flexed and their vertebrae aligned in the sagittal plane. Hind limbs were extended forward with the stifles flexed to help maintain a symmetrical sternal recumbent position. Positioning was confirmed on dorsal and lateral views of a preliminary scan. Subsequently, the spine was imaged from T1-S4 (0.625 mm, 120 kVp, 270 mAs, 40 cm field of view). The lumbosacral region was aseptically prepared with alternating chlorhexidine and 70% isopropanol. A 22 G, 1” needle inserted in the skin to a depth of ½” at approximately L3-L4 using the cranial border of the flexed stifle as a landmark. A second preliminary scan was performed to permit localization of the needle position in the sagittal and dorsal planes and guide a lumbar puncture with a spinal needle (20 G, 3.5”, Becton, Dickson and Company, Franklin Lakes, NJ, USA) that was advanced to the floor of the vertebral canal between L3-L4. Following a subsequent preliminary scan to confirm the position of the spinal needle, a 36” microbore extension set (ICU Medical, Inc., San Clemente, CA) and 20 mL luer tip syringe (Exelint^®^ International, Co. Redondo Beach, CA) pre-filled with contrast medium were attached. Initially, 1.5 mL of iodinated contrast (Ominpaque^™^ Iohexol +PLUSPAK^™^, GE Healthcare, Chicago, IL) [37] was slowly injected and a preliminary scan performed to confirm contrast placement in the subarachnoid space. Once confirmed, the full dose of contrast (3 mL/kg) was injected over 2 minutes followed immediately by exposures of the spine identical to the initial scan. Three-dimensional reconstructions of each spinal column at all time points were created from 2D slices with manual segmentation using commercially available software (Aviso 3D, v2021.1, Thermo Fisher Scientific, Inc., Waltham, MA) for subjective comparisons.

All images were viewed in the bone window using diagnostic image viewing software (OsiriX MD v.8.5.2, Bernex, Switzerland) and morphometric analysis performed according to previously published methods [37]. Briefly, the area for the spinal trauma was localized on three-dimensional reconstructions of individual slices on images obtained pre-op, immediately after surgery as well as 6 and 12 weeks after surgery. Subsequently, vertebral canal (VC) and SC and dural sac (DS) segment measures were performed on 2-D multiplanar reconstructions at the mid-body and intervertebral space of each of three vertebrae cranial and caudal to the cord injury using the software calibrated line measurement tool by the same investigator (CT). Measures included sagittal (height) and transverse (width) diameters of the VC and SC and included three replicate measures per slice. Area measurements were made of the SC, DS, and VC at the mid-body and the intervertebral disc space of each spinal segment, again each measure was repeated three times/slice. Mean and ratios for SC/DS, DS/VC were calculated for each site in all gilts. The presence of contrast filling and complete vertebral body was used to determine which vertebral segments were included in area calculations. Contrast filling around a minimum of 50% or 180° of the spinal cord was required to delineate the margins sufficiently for area measures. The percentage of 2-D slices without detectable contrast between the caudal border of T7 and the cranial border of L2 was also determined. Briefly, the total number of slices with contrast between the two borders was divided by the total number of slices to give a percentage at each time point. All animals met the inclusion criterion and completed the study.

### Sample harvest and magnetic resonance imaging (MRI)

Following humane euthanasia administered intracardiac according to American Veterinary Medicine Association guidelines 12 weeks after surgery (sodium pentobarbital, 200 mg/kg, Vortech Pharmaceuticals, Ltd, Dearborn, MI), the T8-T12 spinal column was harvested intact and surrounding soft tissue removed sufficiently to allow removal of internal fixation. Specimens were then immersed in 10% neutral buffered formalin at a ratio of 2:1 [38]. The fixative was replaced after 24 hr and subsequently every 2–3 days for a minimum of 2 weeks.

For imaging, intact, fixed specimens submersed in saline in sealed plastic bags were placed in ventral recumbency for imaging within an 8-channel rapid wrist coil of a 1.5T MRI unit (Hitachi Echelon, Hitachi Medical Systems America, Inc., Twinsburg, OH) with a 2.5 mm slice thickness and a standard protocol: sagittal T2 FSE, sagittal T1 FSE, dorsal T2 FSE, sagittal fast inversion recovery, and transverse dual phase PD/T2 weighted.

### Histology

Representative cross and longitudinal sections of the formalin fixed spinal cord at the level of, 1 cm cranial and 1 cm caudal to the injury site were embedded in paraffin, sectioned (4 μm) and stained with hematoxylin and eosin. Spinal cord sections at the injury site were also stained with Luxol Fast Blue and Masson’s trichrome to assess for myelin and collagen, respectively, following standard laboratory procedures. The ratio of gray to white matter area on digital images of three consecutive sections within each level (1 cm cranial and caudal to and at the injury site) was quantified on Luxol Fast Blue stained sections with manual segmentation using the scanner graphics software (NanoZoomer S360, Hamamatsu Corporation, Bridgewater, NJ).

### Glial fibrillary acidic protein immunohistochemistry (GFAP)

Immunohistochemistry for glial fibrillary acidic protein (GFAP) was performed in the sections of spinal cord to highlight GFAP+ glial cells which is associated with glial scarring. Briefly, 4 μm sections of formalin fixed paraffin embedded tissues were mounted on positively charged Superfrost^®^ Plus slides (VWR, Radnor PA) and subjected to immunohistochemistry using the automated BOND-MAX and the Polymer Refine Detection kit (Leica Biosystems, Buffalo Grove, IL). Following automated deparaffinization, slides were incubated with a 1:500 dilution of rabbit anti-GFAP antibody (Z0334, Dako, Carpinteria, CA) with no antigen retrieval steps. Sections were incubated with the primary antibody for 30 minutes at room temperature, followed by a polymer-labeled goat anti-rabbit IgG coupled with horseradish peroxidase (Leica Biosystems) for 20 min at room temperature. Subsequently, 3,3’-diaminobenzidine tetrahydrochloride (DAB) was used as the chromogen (10 minutes), and counterstaining was performed with hematoxylin. Slides were mounted with a permanent mounting medium (Micromount^®^, Leica Biosystems).

## Results

All but 2–3 mm of SC compressed between the rod and pedicle was grossly injured, discontinuous and devascularized. All gilts were paraplegic upon recovery from general anesthesia. They exhibited periods of systemic hyperthermia (above 101.4℉) approximately 6 hr after the injury, which resolved with increased fluid administration in combination with pain management. Gilts were largely immobile until 3–5 days post-operatively. However, all gilts in the DRPEG cohort and one from the DPEG cohort could rise to a sitting position between 7 and 14 days after surgery. Beginning 4 to 9 weeks after surgery, unlike the DPEG group the DRPEG gilts could rotate on their hind quarters to change their lateral recumbency. Urinary catheters were maintained for 15 to 20 days until reflex voiding was apparent. Daily urine output was measured at approximately 1.5 and 2mL/kg/hr. Values from serum chemistry and complete blood count remained within normal limits throughout the study period. All pigs had signs of urinary tract infection between 7–10 days post-operatively, and one pig showed leukocytosis 6 weeks after surgery. Urinary tract infections were treated with ethylenediaminetetraacetic acid lavage and procaine penicillin G, 1.4x10^3^ IU/kg, IM, q24 hr for 7 days (VetriPen G^™^, VetOne^®^, Boise, ID), amikacin, 15 mg/kg IM BID for 7 days (Amiglyde-V^®^, Zoetis, Inc, Kalamazoo, MI) or enrofloxacin, 5 mg/kg IM q24 hr for 10–14 days, based on bacterial culture results. The leukocytosis was assumed to be from a urinary tract infection given lack of other signs and since it resolved with 10 days of enrofloxacin at 5 mg/kg IM q24 hr.

### Sensory and motor function

**Fig 2:**
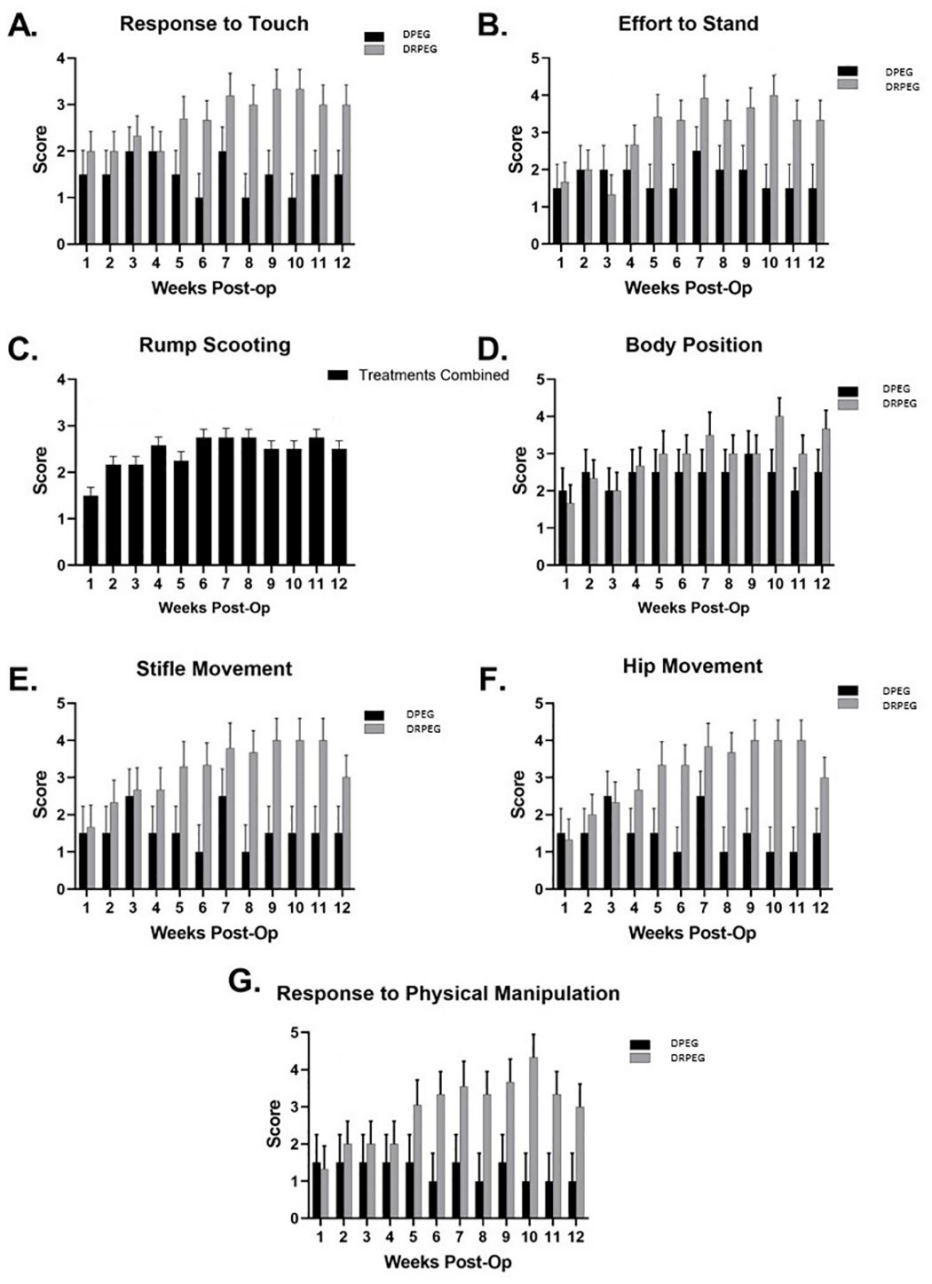
Sensory and motor outcomes including response to touch (A), effort to stand (B), rump scooting (C), body position (D), stifle movement (E), hip movement (F) and response to manipulation (G).

Significant improvements in effort to stand and response to physical manipulation were evident in the DRPEG cohort with increasing time post-surgery (Fig 2), specifically at 10 weeks point compared to the first week. The response to physical manipulation was also higher at 9 and 10 weeks compared to the first week. Similar improvement was observed for the stifle and hip movement. In the DPEG group no improvement was observed at the end of the 12^th^ week with fluctuations throughout the follow up period. With treatment cohorts combined, the rump scooting score gradually improved from the first to the 12^th^ week of follow up (S1 File).

### Computed tomography (CT)

**Fig 3:**
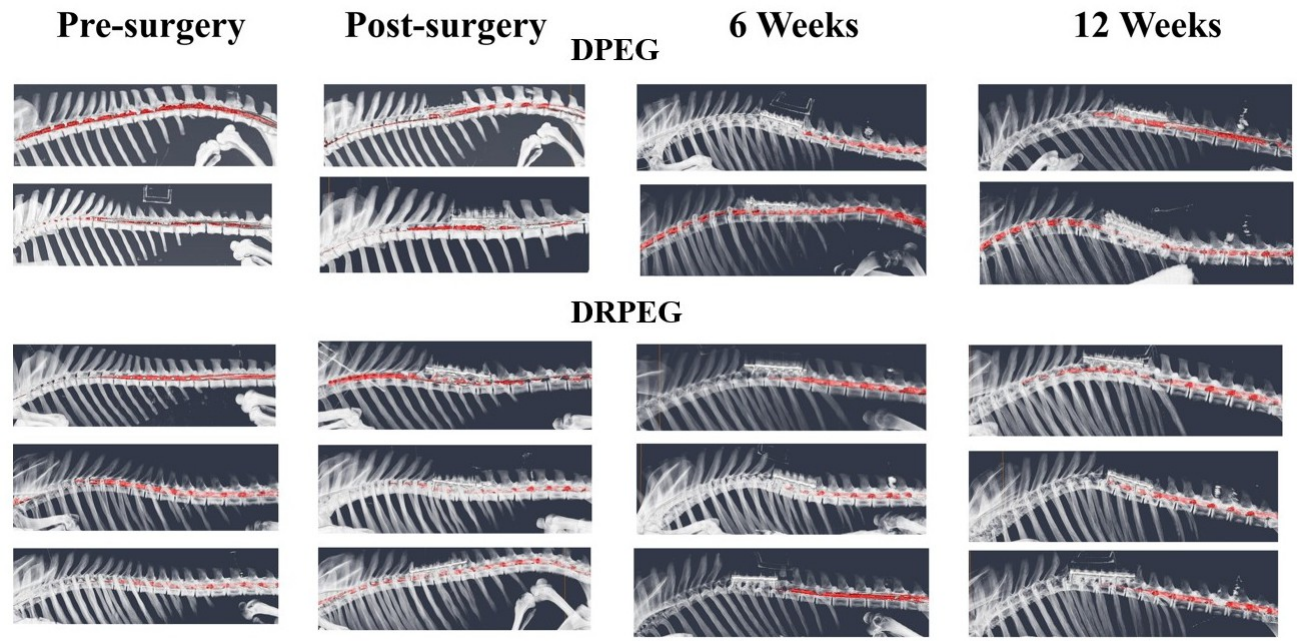
Three-dimensional computed tomography myelogram reconstructions (left view) of gilts with (reconstructed) and without (untreated) sural nerve spinal cord reconstruction before (pre-surgery), immediately after (post-surgery), and 6 and 12 weeks after impact injury. Myelogram contrast (red) was manually segmented on 2-dimensional computed tomography slices.

All pre-surgery myelograms were within normal limits (Fig 3). Immediately after laminectomy, contrast was evident cranial to the injury site, though there were contrast voids at the level of the injury site. Lack of contrast beyond the point of injury in 5/6 of the gilts confirmed dural adhesions 6 weeks after injury. One gilt in the DPEG cohort had a defect at the injury site, and there was poorly delineated contrast cranial to the injury. Twelve weeks after surgery, contrast fill was attenuated cranial to the injury site in all gilts.

**Fig 4:**
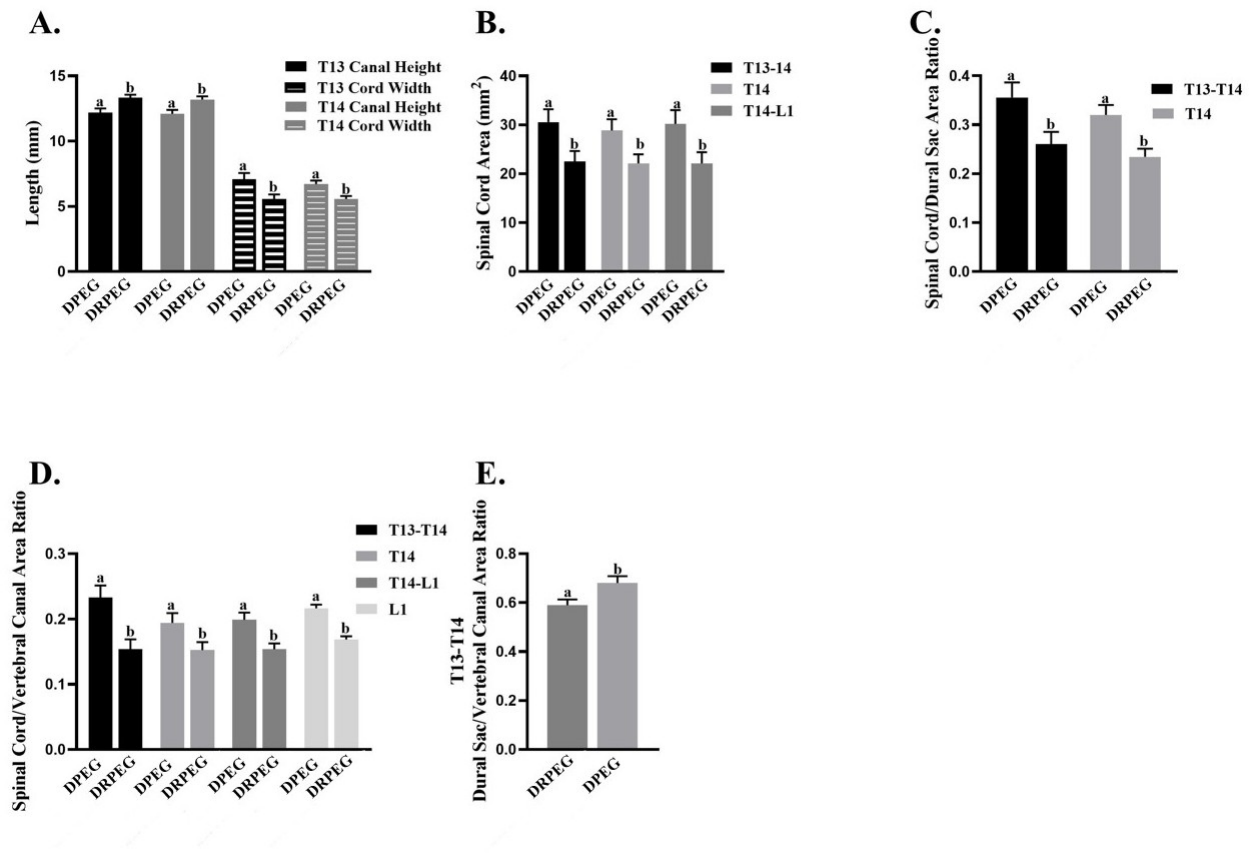
Spinal column measures performed on 2-dimensional computed tomography images with time points combined including canal height and cord width (A); spinal cord area (B); spinal cord/dural sac area ratio (C); spinal cord/vertebral canal area ratio (D) and dural sac/vertebral canal area ratio. Levels are indicated on each graph.

**Fig 5:**
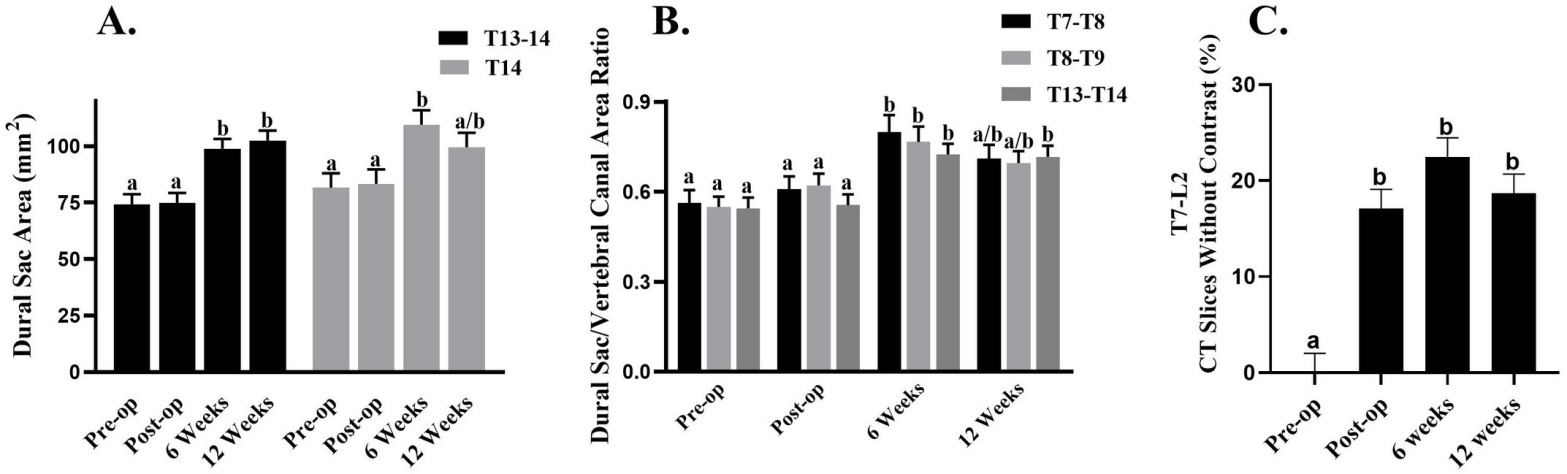
Measures on 2-dimensional spinal column computed tomography images before (pre-op), immediately after (post-op) and up to 12 weeks after injury with treatment groups combined including dural sac area (A), dural sac/vertebral canal area ratio (B) and percentage of CT slices between T7 and L2 without contrast (C).

With all time points combined, differences among CT myelogram measures included greater T13 and T14 vertebral canal height and lower spinal cord width in the DRPEG cohort versus DPEG (Fig 4A). The T13-14, T14, and T14-L1 spinal cord areas were greater in the DPEG cohort (Fig 4B). Both the T13-T14 and the T14 spinal cord/dural sac area ratios were greater in the DPEG cohort (Fig 4C). Similarly, T13-14, T14, T14-L1, and L1 spinal cord/vertebral canal area ratios in the DPEG cohort were greater than those in the DRPEG (Fig 4D), and the dural sac to vertebral canal area ratio was greater in the DPEG cohort at T13-14 with time points combined (Fig 4E). With treatments combined, the T13-T14 dural sac areas were greater 6 and 12 weeks after the surgery relative to pre-() and post-surgery (Fig 5A). The T14 dural sac area was greater 6 weeks after surgery versus pre-surgery (Fig 5A). The T13-T14 dural sac/vertebral canal area ratio was higher 6 and 12 weeks after surgery compared to pre- (Fig 5B) and immediately post-surgery, and the T7-T8 and T8-T9 dural sac/vertebral canal area ratios were both greater 6 weeks after surgery relative to pre-surgery. The percentage of 2D slices with no visible contrast was greater before versus immediately, 6, and 12 weeks after surgery (Fig 5C).

### Magnetic resonance imaging

**Fig 6:**
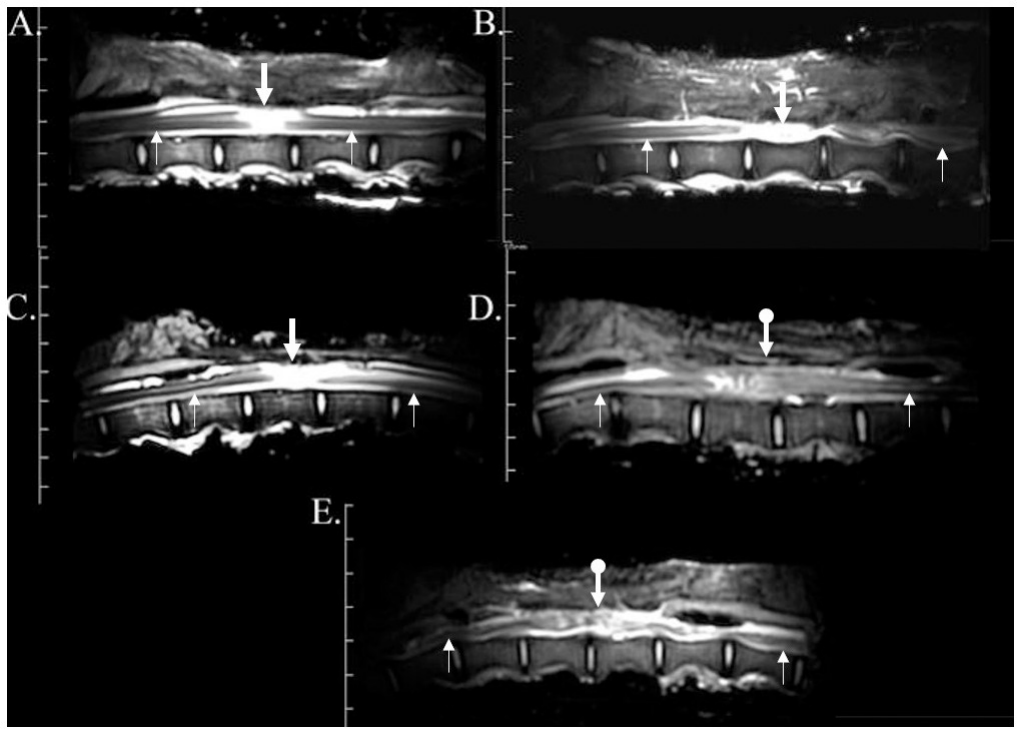
Post-mortem magnetic resonance myelogram on isolated spinal columns of gilts without (A,B) and with (C-E) sural nerve spinal cord reconstruction following blunt impact injury 12 weeks after surgery. None of the controls and two of the reconstructed spinal cord MRI images showed some continuity of the cord (downward arrows with circle). Thin upward arrows point to the intact spinal cord proximal and distal to the area of injury and the thick downward arrows point to the area of discontinuity of the spinal cord.

Twelve weeks after surgery, DPEG group showed severe discontinuity of the spinal cord on the MRI myelogram (Fig 6A and 6B). In the DRPEG cohort 2 out of 3 showed partial continuity (1–2 MRI sequences on the sagittal view of the neural tissue at the area of the blunt trauma with inflammation (Fig 6D and 6E), while one did not (Fig 6C).

### Histology and glial fibrillary acidic protein immunohistochemistry (GFAP)

**Fig 7:**
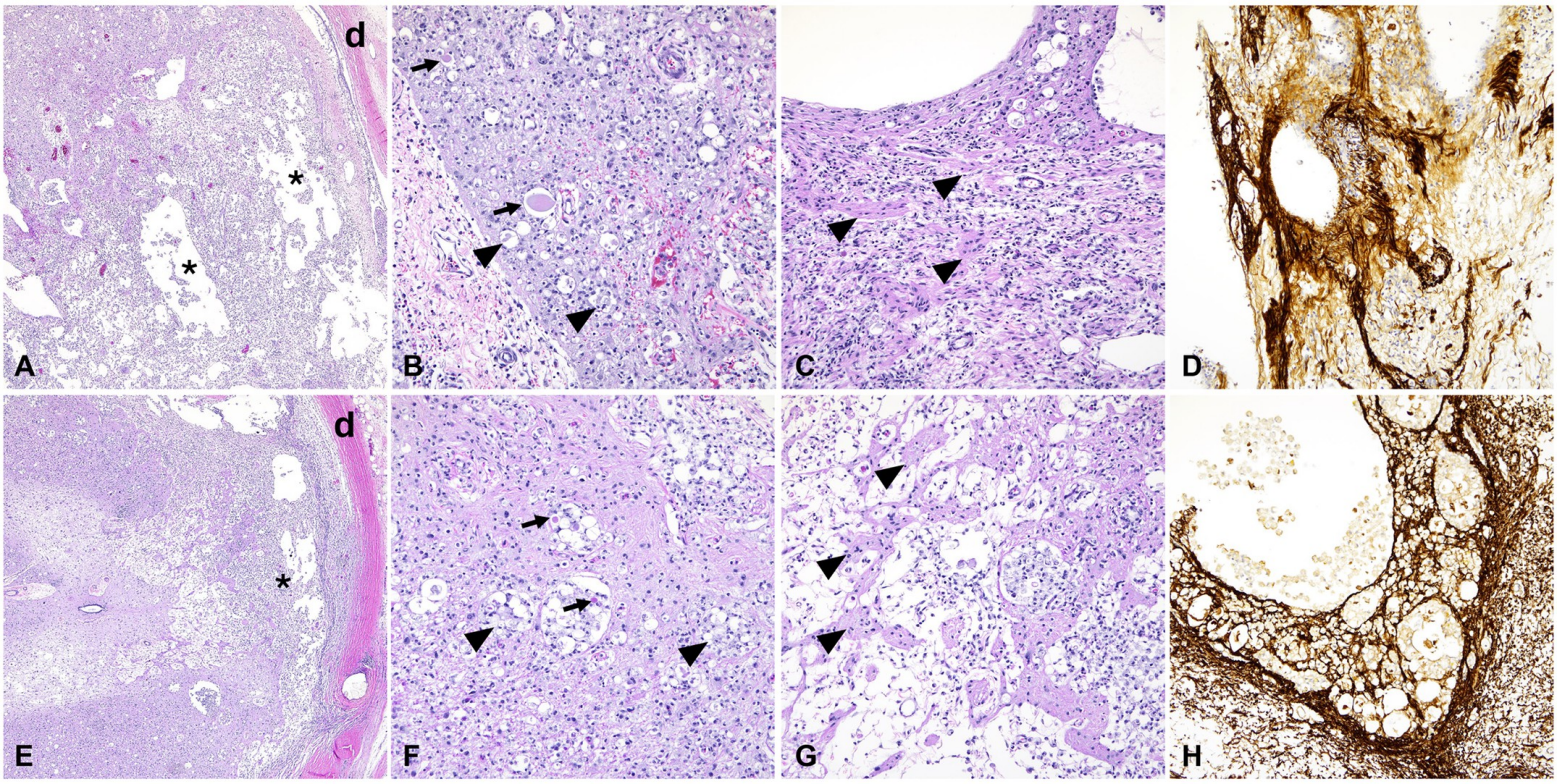
Histological alterations in the spinal cord of pigs subjected to laminectomy (A-D) or laminectomy with spinal cord reconstruction (E-H). Histological changes were similar between the two groups. From low magnification, there are extensive areas of necrosis with loss of neuroparenchyma and cavitation (A and E, asterisks; d: dura mater). There is extensive axonal degeneration (B and F) with spheroids (arrows) and digestion chambers composed of axonal debris and gitter cells (arrowheads). Affected areas also show reactive gliosis (C and G, arrowheads) with intense GFAP immunolabeling (D and H). Stain: H&E (A, B, C, E, F and G), GFAP immunohistochemistry (D and H). Magnification: 50x (A, E), 200X (B, C, D, F, G, H).

The histologic changes at the level of the injury site were severe and similar in all specimens evaluated (n = 5), with histologic alterations of progressively less severity noted cranially and caudally to the injury site, respectively. At the injury site, histologic alterations in the spinal cord included extensive and severe axonal (Wallerian) degeneration, necrosis (myelomalacia), loss, and replacement by reactive glial proliferation (Fig 7). Specifically, myelinated axons in the white matter showed numerous digestion chambers containing axonal and myelin debris, axonal swelling (spheroid formation) and fragmentation, and intense inflammation that segmentally effaced both the white and grey matter with perivascular cuffs of lymphocytes and plasma cells and reactive glial proliferation. Though not significant, the ratio of gray to white matter tended to be higher in the DRPEG (0.21 ± 0.03) compared to the DPEG (0.16 ± 0.03) cohort. At the level of the injury, GFAP+ glial cells heavily surrounded areas of necrosis/parenchymal loss and were the main component of areas of repair (Fig 7).

Sections cranial to the injury site were mostly characterized by axonal degeneration within the white matter, with digestion chambers, axonal swelling, and less intense gitter cell infiltration. The grey matter contained multiple chromatolytic to necrotic neuronal bodies. Sections caudal to the injury site showed evidence of axonal degeneration with digestion chambers and reactive glial cells, but of significantly reduced severity when compared to the injury site and sections obtained cranial to it.

## Discussion

In this study, the efficacy of spinal cord reconstruction using sural nerve autograft and PEG after SCI resulting from blunt trauma was evaluated using a previously established porcine model [18]. Spinal cord injuries usually happen after fractures of the spine following blunt trauma. Currently, the spinal cord cannot be repaired with current surgical techniques. The standard of treatment includes decompression and stabilization of the spine and hemodynamic stabilization of the patient [39]. This is the first study showing significant improvement of the neural function using a novel reconstruction after a blunt complete SCI. Results showed that using nerve graft in the injured area of the spinal cord can provide significant neurological recovery. Although the histological findings showed inflammation and necrosis in both groups the neurological recovery and continuity of the cord (on the MRI) showed superiority in the DRPEG cohorts. The larger spinal cord/dural sac ratio DRPEG cohort indicates less severe atrophy of the cord. Taken together, results suggest recovery in the DRPEG treatment cohort to a level of incomplete spinal cord injury, confirming use of this model in adults versus juvenile swine. We chose T9-T10 for the spinal cord injury model for the ease of access since it’s caudal to the scapula and the spinous processes are not as long as the more cranial thoracic vertebras. The presence of rib cage gives these segments more stability after the laminectomy as opposed to the more caudal segments. In Lee et al. study the pigs were followed for 12 weeks to capture any possible delayed recoveries [18].

A previous study [18] showed that dropping a 100 gr rod from 50 cm followed by 5 minutes of continuous pressure on the cord without reconstruction lead to severe paralysis of the hind limbs with little improvement over the course of 12 weeks consistent with results of the DPEG cohort. Pathological changes of the thoracolumbar spine were evaluated using a previously published CT- guided myelography technique [37]. Results showed significant pathological changes to the SC area and SC/DS/VC ratios distal to the site of injury between DPEG and DRPEG, confirming findings from a previous study that reported high coefficient of variation in in spinal cord area and SC/DS/VC ratios in unaffected animals, with the lowest variation seen in DS/VC canal ratios [37]. Most interesting, in this pilot study, DRPEG treatment resulted in significantly lower variation compared to the DPEG animals indicating reduced cord swelling distal to the injury. It is reported that severity of injury affects residual deformation following injury, with larger cord deformation being found in animals with moderate severity injuries [40]. Similar SCI studies have shown that severe SCI led to atrophic changes of the spinal cord distal to the lesion) [41] and cause lumbar motor neurons remote to the lesion to receive less synaptic input thereby exhibiting lower neuronal activity [41]. A narrow dural sac area in the L1/L2 to L4/L5 on MRI has been associated with the presence of lower back pain in humans [42]. Studies have attempted to correlate imaging results with neurologic outcomes in patients with acute SCI [43–45] and it has been reported that complete SCI causes the largest spinal cord edema area at 50%, while incomplete SI results in approximately 33%. It is reported that no more than 0.9% of patients fully recover from complete or incomplete SCI and many suffer from progressive neurologic deterioration, progressive cord compression or tethering [46]. Continuity of the SC evident with MRI in 2/3 gilts in the reconstruction cohort may indicate some incorporation of the sural autograft into spinal cord tissue. While MRI is used as a standard for neurophysiological evaluation, MRI alone does not show the functional status of the cord [47]. The spinal cord and dural sac ratio may have implications for the severity and prognosis of spinal cord injuries. A narrower SC and DS ratio may translate to a smaller buffer of cerebrospinal fluid (CSF) around the SC, which provides a protective layer for the SC and thereby reducing the risk of compression and injury. On the other hand, a larger ratio suggests a bigger CSF ratio which would provide increased protection for the SC. In case of injury, a larger buffer may mitigate the extent of damage and potentially contribute to better recover prospects. In this study, less severe atrophy of the cord in the DRPEG cohort was indicated by a larger spinal cord/dural sac ratio and dural sac area compared to the DPEG. It is worth noting that changes in the diameter of the canal and dural space may be related to the laminectomy procedure and adhesions of the dura after surgery.

Sharp spinal cord injuries can provide an insight to understanding the process of spinal cord recovery. After lumbar spinal cord transection in lizards, a bridge tissue representing the regenerated cord forms between the proximal and distal ends. The bridge tissue comprises of nervous tissues, glial cells, nerves, and sparse fibrocytes from the meninges. In the regenerating tail a thin spinal cord forms from the tail stump, which consists of an ependymal tube surrounded by a thin rim of axons [48–51]. This pattern is similar to the peripheral nerve injuries where the axons sprout for the proximal end to bridge the gap. In order to guide the growth of the proximal end conduits or nerve auto or allografts are used to reconstruct the peripheral nerves in humans [52]. A similar concept for the reconstruction of the SC was used in this porcine model of SCI study. The goal of reconstruction is to restore the function of spinothalamic and corticospinal tracts to improve the motor and sensory function of the lower extremities. It is reported that severity of injury affects residual deformation following injury, with larger cord deformation being found in animals with moderate severity injuries [40]. A similar concept for the reconstruction of the SC was used in this porcine model of SCI study. The goal of reconstruction is to restore the function of spinothalamic and corticospinal tracts to improve the motor and sensory function of the lower extremities. It is reported that severity of injury affects residual deformation following injury, with larger cord deformation being found in animals with moderate severity injuries lower neuronal activity [41]. The extent of tissue inflammation was extensive in both cohorts at 3 month follow up with no histological differences being observed. Both cohorts showed inflammation, demyelination, necrosis, and reactive glial proliferation; these findings that are expected after neural injury [53]. The acute phase of spinal cord injury includes vascular damage and free radical formation, which lead to apoptosis and demyelination in the subacute phase. Cystic cavities and glial scar subsequently form in the chronic phase [3]. GFAP indicates transformation of normal astrocytes into reactive astrocytes which results in glial scar formation. These reactive astrocytes are thought to have detrimental effects of spinal cord injury repair [54]. This model illustrated that the extent of neurological recovery correlate with the extent of spared white and grey matter. Histology can reveal changes caused by trauma; several factors may limit the visibility of such evidence on the spinal cord. The healing process of tissue after blunt force trauma can influence histological findings, such that the extent of injury is no longer evident. While other outcome assessments in this study show recovery, histological evidence did not support differences between groups and were consistent with severe SCI and effective transection.

David and Aguayo investigated the extent of axonal growth following localized central nervous system injury in adult rats. By utilizing peripheral nerve segments as connectors between the medulla and spinal cord, axons from neurons at these levels extended around 30 millimeters [55]. There are a variety of nerves described in pigs which are amenable to harvesting. These include sural, ulnar, facial and laryngeal nerves that are easily accessible and cause minimal functional loss after such harvests [56]. The sural nerve (9–10 cm) provides the largest length followed by the ulnar nerve [57]. The sural nerve was used to preserve and/or restore complete function of the limbs as it is one of the longest nerves with ease of access, mitigating blood loss [47]. Similar to DRPEG groups, previous reports have shown that peripheral nerve grafts for SCI can provide improved functional and sensory recovery even in incomplete injuries [47]. The DPEG group used as the control group had motor and sensory results similar to those in previous studies [11].

Multiple families of inorganic polymers can immediately fuse damaged cell membranes and seal the endothelium after mechanical injury [21]. In this study, PEG was used to improve incorporation of the sural autograft with the spinal cord. PEG molecules are equally effective independent of the weight and have been shown to be nontoxic, water-soluble, and are safe for use man [58, 59]. When two adjacent cell membranes touch in the presence of PEG, they fuse and cytoplasms mix. Acute dehydration of the plasmalemmas is followed by the fusion of the outer and then the inner membrane leaflets [25]. PEG has been reported to improve the survival of the neural cells in previous studies [21, 60–63] and was used as the fusogen for both DRPEG and DPEG groups. Use of a fusogen alone did not show any significant improvement in the DPEG group. The function of Fusogen is sealing the membrane of the neural cells and in the presence of normal neural tissue (cranial end of the SC and sural nerve autograft in the DRPEG cohort) it could provide better neurological recovery. However, when Fusogen (PEG) was used in the control group between the cranial end of the SC and damaged and devascularized neural tissue caudally the extent of neurological recovery was far less.

This pilot study was limited by the number of animals in each treatment group, as the main goal was to refine techniques and test proof of concept. The paralyzed animals required diligent care postoperatively which is time consuming and costly. Although the standard of care is decompression and fusion for spinal cord injuries, this study shows that reconstruction of the spinal cord may improve neurological function. We did not measure the impact force each time the rod dropped but the rod and the height from which it was dropped were consistent and the device was attached to the table. Future studies need to be directed at the use of other nerve grafts, and possible simultaneous use of other modalities such as ultrasound or pharmacological interventions to increase the magnitude of recovery in spinal cord injuries.

## Conclusion

This study showed promising results for the use of sural nerve autograft and PEG for reconstruction of the spinal cord after blunt trauma in a large animal model. Future work should focus on the use of other nerve graft, fusogens or stems cells to improve the neurological function after spinal cord injury. Furthermore, exploring the relationship between the SC and DS ratio, degree of trauma, and subsequent recovery trajectory may allow for clinicians to implement this ratio as of several factors to assess injury severity and predict potential outcomes.

## Supporting information

S1 File Data regarding the clinical assessment of the pigs. (DOCX)
