# Supplementary material for: A novel reconstruction model for thoracic spinal cord injury in swine
Source: PLoS One. 2024 Sep 26;19(9):e0308637. doi: 10.1371/journal.pone.0308637 (PMC11426471; doi:10.1371/journal.pone.0308637)
Supplement: S1 File — (DOCX) [file pone.0308637.s001.docx]

|  | Week | Treatment | Neurofunction | Level of sensation (knee, Calx, etc) | Active movement of the hips (Flexion and/or extension) | Active movement of the knees(Flexion and/or extension) | Neurofunction | Effort to stand up | Able to use the hind limbs to scoot over | Body Position | Physiotherapy Resistance | Neurofunction | Bladder Function |
| --- | --- | --- | --- | --- | --- | --- | --- | --- | --- | --- | --- | --- | --- |
| Pig 1603 | 1 | Reconstructed | sensory | 2 | 1 | 2 | Motor | 1 | 1 | 1 | 1 | Sphincter | No |
| Pig 1603 | 2 | Reconstructed | sensory | 2 | 1 | 2 | Motor | 1 | 2 | 1 | 2 | Sphincter | Yes |
| Pig 1603 | 3 | Reconstructed | sensory | 3 | 3 | 4 | Motor | 1 | 3 | 1 | 3 | Sphincter | Yes |
| Pig 1603 | 4 | Reconstructed | sensory | 1 | 2 | 2 | Motor | 3 | 3 | 4 | 2 | Sphincter | Yes |
| Pig 1603 | 5 | Reconstructed | sensory | 3 | 4 | 4 | Motor | 4 | 3 | 4 | 3 | Sphincter | Yes |
| Pig 1603 | 6 | Reconstructed | sensory | 3 | 4 | 4 | Motor | 4 | 3 | 4 | 3 | Sphincter | Yes |
| Pig 1603 | 7 | Reconstructed | sensory | 3 | 4 | 4 | Motor | 4 | 3 | 4 | 3 | Sphincter | Yes |
| Pig 1603 | 8 | Reconstructed | sensory | 3 | 4 | 4 | Motor | 3 | 3 | 3 | 3 | Sphincter | Yes |
| Pig 1603 | 9 | Reconstructed | sensory | 3 | 4 | 4 | Motor | 3 | 3 | 4 | 3 | Sphincter | Yes |
| Pig 1603 | 10 | Reconstructed | sensory | 3 | 3 | 3 | Motor | 3 | 3 | 4 | 3 | Sphincter | Yes |
| Pig 1603 | 11 | Reconstructed | sensory | 3 | 3 | 3 | Motor | 3 | 3 | 2 | 3 | Sphincter | Yes |
| Pig 1603 | 12 | Reconstructed | sensory | 3 | 2 | 2 | Motor | 2 | 3 | 3 | 2 | Sphincter | Yes |
| Pig 1605 | 1 | Reconstructed | sensory | 1 | 1 | 1 | Motor | 1 | 1 | 3 | 1 | Sphincter | No |
| Pig 1605 | 2 | Reconstructed | sensory | 1 | 1 | 1 | Motor | 2 | 2 | 3 | 1 | Sphincter | Yes |
| Pig 1605 | 3 | Reconstructed | sensory | 2 | 2 | 2 | Motor | 1 | 2 | 2 | 1 | Sphincter | Yes |
| Pig 1605 | 4 | Reconstructed | sensory | 2 | 2 | 2 | Motor | 1 | 2 | 2 | 1 | Sphincter | Yes |
| Pig 1605 | 5 | Reconstructed | sensory | 2 | 2 | 2 | Motor | 2 | 2 | 2 | 2 | Sphincter | Yes |
| Pig 1605 | 6 | Reconstructed | sensory | 2 | 2 | 2 | Motor | 2 | 3 | 2 | 2 | Sphincter | Yes |
| Pig 1605 | 7 | Reconstructed | sensory | 3 | 3 | 3 | Motor | 3 | 3 | 3 | 3 | Sphincter | Yes |
| Pig 1605 | 8 | Reconstructed | sensory | 3 | 3 | 3 | Motor | 3 | 3 | 3 | 2 | Sphincter | Yes |
| Pig 1605 | 9 | Reconstructed | sensory | 3 | 4 | 4 | Motor | 4 | 3 | 2 | 3 | Sphincter | Yes |
| Pig 1605 | 10 | Reconstructed | sensory | 3 | 4 | 4 | Motor | 4 | 3 | 4 | 5 | Sphincter | Yes |
| Pig 1605 | 11 | Reconstructed | sensory | 3 | 4 | 4 | Motor | 3 | 3 | 3 | 2 | Sphincter | Yes |
| Pig 1605 | 12 | Reconstructed | sensory | 3 | 3 | 3 | Motor | 3 | 3 | 4 | 2 | Sphincter | Yes |
| Pig 1607 | 1 | Reconstructed | sensory | 3 | 2 | 2 | Motor | 3 | 1 | 1 | 2 | Sphincter | No |
| Pig 1607 | 2 | Reconstructed | sensory | 3 | 4 | 4 | Motor | 3 | 3 | 3 | 3 | Sphincter | Yes |
| Pig 1607 | 3 | Reconstructed | sensory | 2 | 2 | 2 | Motor | 2 | 2 | 3 | 2 | Sphincter | Yes |
| Pig 1607 | 4 | Reconstructed | sensory | 3 | 4 | 4 | Motor | 4 | 3 | 2 | 3 | Sphincter | Yes |
| Pig 1607 | 5 | Reconstructed | sensory |  |  |  | Motor |  |  |  |  | Sphincter |  |
| Pig 1607 | 6 | Reconstructed | sensory | 3 | 4 | 4 | Motor | 4 | 3 | 3 | 5 | Sphincter | Yes |
| Pig 1607 | 7 | Reconstructed | sensory |  |  |  | Motor |  |  |  |  | Sphincter |  |
| Pig 1607 | 8 | Reconstructed | sensory | 3 | 4 | 4 | Motor | 4 | 3 | 3 | 5 | Sphincter | Yes |
| Pig 1607 | 9 | Reconstructed | sensory | 4 | 4 | 4 | Motor | 4 | 3 | 3 | 5 | Sphincter | Yes |
| Pig 1607 | 10 | Reconstructed | sensory | 4 | 5 | 5 | Motor | 5 | 3 | 4 | 5 | Sphincter | Yes |
| Pig 1607 | 11 | Reconstructed | sensory | 3 | 5 | 5 | Motor | 4 | 3 | 4 | 5 | Sphincter | Yes |
| Pig 1607 | 12 | Reconstructed | sensory | 3 | 4 | 4 | Motor | 5 | 3 | 4 | 5 | Sphincter | Yes |
| Pig 1456 | 1 | control | sensory | 2 | 2 | 2 | Motor | 2 | 2 | 3 | 2 | Sphincter | No |
| Pig 1456 | 2 | control | sensory | 2 | 2 | 2 | Motor | 2 | 2 | 2 | 2 | Sphincter | Yes |
| Pig 1456 | 3 | control | sensory | 3 | 4 | 4 | Motor | 2 | 2 | 2 | 2 | Sphincter | Yes |
| Pig 1456 | 4 | control | sensory | 3 | 2 | 2 | Motor | 2 | 3 | 2 | 2 | Sphincter | Yes |
| Pig 1456 | 5 | control | sensory | 2 | 2 | 2 | Motor | 1 | 2 | 2 | 2 | Sphincter | Yes |
| Pig 1456 | 6 | control | sensory | 1 | 1 | 1 | Motor | 1 | 2 | 2 | 1 | Sphincter | Yes |
| Pig 1456 | 7 | control | sensory | 3 | 3 | 4 | Motor | 3 | 3 | 2 | 2 | Sphincter | Yes |
| Pig 1456 | 8 | control | sensory | 1 | 1 | 1 | Motor | 2 | 2 | 2 | 1 | Sphincter | Yes |
| Pig 1456 | 9 | control | sensory | 2 | 2 | 2 | Motor | 2 | 2 | 3 | 2 | Sphincter | Yes |
| Pig 1456 | 10 | control | sensory | 1 | 1 | 2 | Motor | 1 | 2 | 2 | 1 | Sphincter | Yes |
| Pig 1456 | 11 | control | sensory | 2 | 1 | 2 | Motor | 2 | 3 | 2 | 1 | Sphincter | Yes |
| Pig 1456 | 12 | control | sensory | 2 | 2 | 2 | Motor | 2 | 2 | 2 | 1 | Sphincter | Yes |
| Pig 1463 | 1 | control | sensory | 1 | 1 | 1 | Motor | 1 | 2 | 1 | 1 | Sphincter | No |
| Pig 1463 | 2 | control | sensory | 1 | 1 | 1 | Motor | 2 | 2 | 3 | 1 | Sphincter | Yes |
| Pig 1463 | 3 | control | sensory | 1 | 1 | 1 | Motor | 2 | 2 | 2 | 1 | Sphincter | Yes |
| Pig 1463 | 4 | control | sensory | 1 | 1 | 1 | Motor | 2 | 2 | 3 | 1 | Sphincter | Yes |
| Pig 1463 | 5 | control | sensory | 1 | 1 | 1 | Motor | 2 | 2 | 3 | 1 | Sphincter | Yes |
| Pig 1463 | 6 | control | sensory | 1 | 1 | 1 | Motor | 2 | 3 | 3 | 1 | Sphincter | Yes |
| Pig 1463 | 7 | control | sensory | 1 | 2 | 1 | Motor | 2 | 2 | 3 | 1 | Sphincter | Yes |
| Pig 1463 | 8 | control | sensory | 1 | 1 | 1 | Motor | 2 | 3 | 3 | 1 | Sphincter | Yes |
| Pig 1463 | 9 | control | sensory | 1 | 1 | 1 | Motor | 2 | 2 | 3 | 1 | Sphincter | Yes |
| Pig 1463 | 10 | control | sensory | 1 | 1 | 1 | Motor | 2 | 2 | 3 | 1 | Sphincter | Yes |
| Pig 1463 | 11 | control | sensory | 1 | 1 | 1 | Motor | 1 | 2 | 2 | 1 | Sphincter | Yes |
| Pig 1463 | 12 | control | sensory | 1 | 1 | 1 | Motor | 1 | 2 | 3 | 1 | Sphincter | Yes |
